# Dr. Taft on Dentition

**Published:** 1850-10

**Authors:** J. Taft


### DR. TAFT ON DENTITION.

A DISSERTATION READ BEFORE THE MISSISSIPPI VALLEY ASSOCIATION OF DENTAL SURGEONS, AT CINCINNATI, SEPTEMBER 11th, 1850.

---

BY J. TAFT, D. D. S.

---

*Gentlemen:*—By the appointment of your honorable body it has been my duty to prepare, and now is my privilege to present to you the following brief essay, and I cannot do less than crave your kind indulgence for its imperfections.

The development of the human system is a subject, the investigation of which, at all times, is interesting to the scientific, and highly important to all; fraught with interest, which all who understand must appreciate; whether viewed as one harmonious whole, blended together in mysterious concert, or as separate and distinct organs arising co that exalted function designed by the author of nature.

Here we find ample scope in which the mightiest minds may go forth to the work fully assured of the abundance and richness of the material upon which to operate. We will now for a few moments direct our consideration to the development of the teeth, an organism of momentous import, whether considered alone or in connection with other organs.

A subject, though not altogether overlooked by physiological writers, yet the sterility of their writings upon this subject manifests a great want of thought and investigation in relation to it; there are, however a few noble exceptions.

We cannot for a moment indulge censure or reflection; for the limited investigation which this subject has received at the hands of a majority of writers, has not resulted from its insignificance; but there are so many and such a variety of subjects of paramount importance, such as the fundamental laws of physiology, and those pathological conditions which involve in difficulty the human economy at any and all periods of its existence, that dentition has but here and there received a passing notice.

The physiologist finds here an extended field for research and investigation.

The pathologist, too, finds here a fruitful field in which to labor, controlling those pathological conditions so frequently manifested during the development of the dental organism.

We will confine the following remarks to one or two points in dentition; yet these within the limits of the present essay can only receive a few cursory remarks. The rudiments of the temporary teeth are apparent at a very early period of fœtal existence, probably within forty days after intra-uterine conception; it is quite certain that their formation occurs at as early a period as the distinctive formation of the contiguous parts. This is predicated upon the nature of those parts; these rudiments when first perceptible are minute points or follicules exhibited within a slight groove upon the embryo jaw; this groove developes itself gradually until the germ is enveloped in its little cell, so that before ossification of the germ takes place, it is encased in a bony receptacle, which is formed for its protection during its growth; the gums oftentimes sustain compression which would injure and frequently destroy the germ if it was shielded by the gum only.

There are very many interesting points here for investigation, such as the ossification of the teeth, the manner in which it occurs, the growth of the teeth as the ossific material is supplied to their structure, the development of the enamel, the crusta petrosa, &c., &c. Passing by all of these we will indulge a few remarks upon the eruption of the teeth.

There is a gradual increased capacity of the receptacles of the rudiments of the teeth, which begins prior to the ossification of the crowns. This enlarged capacity becomes necessary to accommodate the growth of the teeth; this is not accomplished by the active agency of the teeth as some suppose, but by the absorbents in anticipation of the growth of the tooth.

Some represent the teeth as accomplishing their eruption, by the force or pressure they exert upon the gum, and that they only escape from their receptacles by force, and that there must necessarily be an abnormal condition produced before this natural process can be accomplished. Is this in accordance with the acknowledged laws of physiology?

Now, I suppose when there is an equal and undisturbed development throughout the system, that each and every organ will perfectly develope itself without any irritation whatever; irritation and disease is only a necessary consequent upon dentition, in so far as nature's laws have been transgressed; when the equilibrium of the system has been disturbed, dentition is a very vulnerable point upon which to manifest itself. Dentition is a delicate process, the perfect exhibition of which is easily intercepted, and hence we should exercise the utmost care lest we interfere with nature's arrangements.

The manner in which the teeth make their exit through the gum, is a process upon which there has been much speculation. It is maintained by some, that it is effected by the elongation of the root; that the bottom of the socket is a fixed point, and as the root elongates the crown consequently pierces the gum and effects its way through it. Blandin mentions an opinion but slightly different from this; he says, "The cause of the eruption of the teeth is very simple, and very apparent; they issue from their alveoli because they have attained to such a size that they can be no longer contained in them." Admitting these positions, it would follow, that there is pressure both against the gum and at the bottom of the socket by the tooth, the latter of which, if it existed, would produce more serious local difficulties than is often met with; it would be impossible to have pressure against the gum by the tooth, without a corresponding pressure in the socket; so then, if there is inflammation in the gum as a consequence of the pressure of the crowns of the teeth against them, then there would be a corresponding or greater amount of inflammation within the socket, for it is much more highly ramified with nerves and vessels than the gum. That neither the growth of the teeth, nor the elongation of their roots, is the instrumentality by which they make their exit through the gum, appears probable from the following case:

A few months ago I removed from the mouth of a boy, aged about eleven years, the first superior bicuspid of the right side; upon examination I found the crown quite loose, and my first impression was, that the tooth was broken off a little above the neck. After separating the gum with the lancet it dropped out, had no root, and only extended about two lines above the termination of the enamel; the termination was cup-shaped, the concavity about a line and a half deep. This tooth evidently never had any root, for it came away at just about that period of life at which the bicuspid teeth are fully developed; it had attained a perfect protrusion through the gum; exactly imitating its fellow of the opposite side, so far as the development of the crown was concerned; it was at too early a period of life to have formed a root, and then have been destroyed by absorption. Again, the appearance of the termination was altogether unlike that produced by absorption; and, again, there was no diseased condition of the surrounding parts apparent, there was nothing unusual apparent about it until within a short time previous to its removal when it became loose, and slightly irritated the margin of the gum. With the cause of this I have nothing to do now. I only adduce the fact to show that the crown of a tooth may make its way through the gum and assume its proper position altogether independent of the growth of the root.

The theory best sustained by facts and analogy, is that maintained by Delabarre. Dr. Harris speaking of this theory says: "M. Delabarre believes the exit of a tooth from its matrix, and its passage through the alveolus and gums are effected in the same manner as the birth of a child. The sack he regards as the chief agent, and that it is by the contraction of this which is adherent to the neck of the tooth, that the tooth is lifted from its socket, and its neck ultimately brought to a level with the gum." "This," says the Dr., "is the only philosophical and truly plausible explanation that has ever been given of this most curious and interesting operation of the animal economy. And when we take into consideration that the inner membrane of the sack is of a fibro-mucous structure, it is easy to perceive how the advance of a tooth may be effected by the contraction of this enclosure, which is firmly attached to its neck and also to the gum. It is by the contraction of this that the dentition of a tooth is effected."

This appears to be the most rational theory yet advanced—is divested of that hypothetical character possessed by others, and seems to have been based upon facts. Delabarre remarks, "I will endeavor to dissipate the doubts that might necessarily arise from these diversities of opinion, and with scalpel, burine and glass in hand, will give my views of nature as being the only book that cannot lead us astray." Here we see it was by actual observation in dissections that Delabarre fixed his conclusions. All the ordinary attendant phenomena upon the emergence of the teeth can be harmonized with this principle.

It does not conflict with any of the known laws of physiology, as do most of the other hypothetical theories that have been advanced, the tendency of which in their practical results are pernicious. Practical applications based upon theories not established by facts are always uncertain in their effects, and hence the necessity of the correctness of our knowledge of primary principles.

